# Cruxome: a powerful tool for annotating, interpreting and reporting genetic variants

**DOI:** 10.1186/s12864-021-07728-6

**Published:** 2021-06-03

**Authors:** Qingmei Han, Ying Yang, Shengyang Wu, Yingchun Liao, Shuang Zhang, Hongbin Liang, David S. Cram, Yu Zhang

**Affiliations:** 1 Berry Genomics Company Limited, Building 5, Courtyard 4, Shengmingyuan Road, ZGC Life Science Park, Changping District, 102200 Beijing, China; 2 Xian Children’s Hospital, 710003 Xian, China

**Keywords:** Cruxome, Next Generation Sequencing, Mendelian disorders, Variant annotation, Variant interpretation, Whole Exome Sequencing, Natural language processing

## Abstract

**Background:**

Next-generation sequencing (NGS) is an efficient tool used for identifying pathogenic variants that cause Mendelian disorders. However, the lack of bioinformatics training of researchers makes the interpretation of identified variants a challenge in terms of precision and efficiency. In addition, the non-standardized phenotypic description of human diseases also makes it difficult to establish an integrated analysis pathway for variant annotation and interpretation. Solutions to these bottlenecks are urgently needed.

**Results:**

We develop a tool named “Cruxome” to automatically annotate and interpret single nucleotide variants (SNVs) and small insertions and deletions (InDels). Our approach greatly simplifies the current burdensome task of clinical geneticists and scientists to identify the causative pathogenic variants and build personal knowledge reference bases. The integrated architecture of Cruxome offers key advantages such as an interactive and user-friendly interface and the assimilation of electronic health records of the patient. By combining a natural language processing algorithm, Cruxome can efficiently process the clinical description of diseases to HPO standardized vocabularies. By using machine learning, in silico predictive algorithms, integrated multiple databases and supplementary tools, Cruxome can automatically process SNVs and InDels variants (trio-family or proband-only cases) and clinical diagnosis records, then annotate, score, identify and interpret pathogenic variants to finally generate a standardized clinical report following American College of Medical Genetics and Genomics/ Association for Molecular Pathology (ACMG/AMP) guidelines. Cruxome also provides supplementary tools to examine and visualize the genes or variations in historical cases, which can help to better understand the genetic basis of the disease.

**Conclusions:**

Cruxome is an efficient tool for annotation and interpretation of variations and dramatically reduces the workload for clinical geneticists and researchers to interpret NGS results, simplifying their decision-making processes. We present an online version of Cruxome, which is freely available to academics and clinical researchers. The site is accessible at http://114.251.61.49:10024/cruxome/.

**Supplementary Information:**

The online version contains supplementary material available at 10.1186/s12864-021-07728-6.

## Background

Genetic diseases that follow an autosomal dominant, autosomal recessive, X-linked dominant, X-linked recessive or mitochondrial pattern of inheritance are known as Mendelian disorders. [1–3]. Currently, in the order of 7,000–9,600 Mendelian disorders have been recorded by Global Genes (https://globalgenes.org/), Online Mendelian Inheritance in Man (OMIM, https://omim.org/) and Orphadata (http://www.orphadata.org/) databases and approximately 300 new Mendelian phenotypes are updated each year [4]. Of all the Mendelian disorders, approximately 80 % now have a defined genetic cause [5, 6] whereas for the remaining 20 %, the genes and genetic lesions remain unknown [7–9]. Thus, clinical research is ongoing to fully characterize the causative genes, develop a better understanding of the underlying disease mechanisms and, explore potential treatment options [10].

Next-generation sequencing (NGS) has emerged as an innovative tool for medical genetics, and has led to a paradigm shift in medical research and clinical practice [11–13]. With the decreasing cost of sequencing, methods such as whole exome sequencing (WES) have become affordable and are widely used for the diagnosis of Mendelian disorders, with typical positive diagnostic yields of 25–40 % [14, 15]. With the fast development of different NGS techniques, the gap between data yield, quality and gene coverage between platforms is rapidly closing. The challenge now is the ability to systematically analyze the hundreds of thousands of high-quality variant calls (including single nucleotide variants, SNVs, short insertions or deletions, InDels and large copy number variants, CNVs) that are revealed in WES sequencing files [16–19]. Even after rigorous filtering, there are still tens to hundreds of candidate causal variants to be considered [19–22]. Thus, an important step is to choose the appropriate analysis tools to efficiently and precisely mine the causative variants, especially when the analysis team lacks training in the use of sophisticated bioinformatic programs. In addition, secondary confirmatory analyses are also required for verification or support when candidates of causative variation are related to the phenotype.

Several open-source analysis tools for variant annotation and functional effect prediction have been reported including spliceAI [20], ANNOVAR [21], SnpEff [23], PolyPhen-2 [24], CADD [25] and InterVar [26]. For example, SpliceAI is a deep learning-based tool specifically designed to identify splice variants. Combined Annotation Dependent Depletion (CADD) is used to score the deleteriousness of SNV as well as InDel variants in the human genome. Alternatively, InterVar can be used for clinical interpretation of genetic variants using the ACMG/AMP 2015 guidelines [26]. However, almost all of these tools are command line tools that have an unfriendly user interface and require a strong background in bioinformatics to comprehensively analyze the data.

When a set of candidate variants are identified, the aim of follow-up analysis is to establish a strong relationship between the candidate genes and known diseases by using information in the published literature and databases. However, this information is sometimes incomplete or fragmented and distributed differently across many databases, which makes this step very time-consuming and inefficient. There are several reported tools that integrate the various databases and simplify the search. These tools include IPAD (integrated pathway analysis database for systematic enrichment analysis) [27], SIDD (semantically integrated database towards a global view of human disease) [28], VariED (integrated database of gene annotation and expression profiles for variants related to human diseases) [29], DisGeNET (integrated information on human disease-associated genes and variants) [30] and Human Disease Insight (integrated knowledge-based platform for disease-gene-drug information) [31]. However, while useful, these tools only focus on specific applications. Thus, comprehensive integration of different databases for relevant knowledge is urgently needed to increase the yield of positive diagnoses.

Electronic health records (EHRs) have been widely implemented by clinical geneticists and include the patient’s information such as name, age, gender, laboratory test results, phenotypic description, diagnosis and medication details. Almost all tools or databases adopt Human Phenotype Ontology (HPO) as the reference. HPO uses standardized vocabulary for describing phenotypic abnormalities in human disease, drawing on over 13,000 terms and over 156,000 annotations to hereditary diseases (https://hpo.jax.org/) [29, 32]. For clinical geneticists, it is almost impossible to accurately describe all of patient’s phenotype using standard terms, and often the diagnosis records are more colloquial and not directly computationally useful [32, 33]. Benefiting from the development of big data techniques, large-scale EHR data mining has become widely used in data-driven medical studies, clinical decision making, and health management [34–36]. Since the phenotypic description of patients is a critical factor for precise variant interpretation, it is urgent to develop new algorithms to efficiently and accurately transform colloquial descriptions to more standardized vocabulary.

Based on these challenges, we develop Cruxome, an automated and user-friendly tool for variant interpretation which is designed to efficiently and precisely handle the Variant Call Format (VCF) file (either from WES or gene panel data) and generate standardized clinical reports. By mining the hundreds of thousands of literature accounts and integrating appropriate databases, Cruxome harbors a comprehensive and regularly updated biomedical knowledge base to keep pace with precise variant interpretation. Cruxome uses a natural language processing algorithm (NER) to transform colloquial descriptions of phenotype to standard HPO vocabulary. Cruxome also supports building a personal knowledge base to efficiently manage patient’s information and interpret results with traceable evidence record of interpretation decisions. Above all, Cruxome provides an overall solution for variant interpretation, dramatically reducing workload and facilitating better decision-making processes.

## Implementation

### Construction of Cruxome and main features

**Fig. 1:**
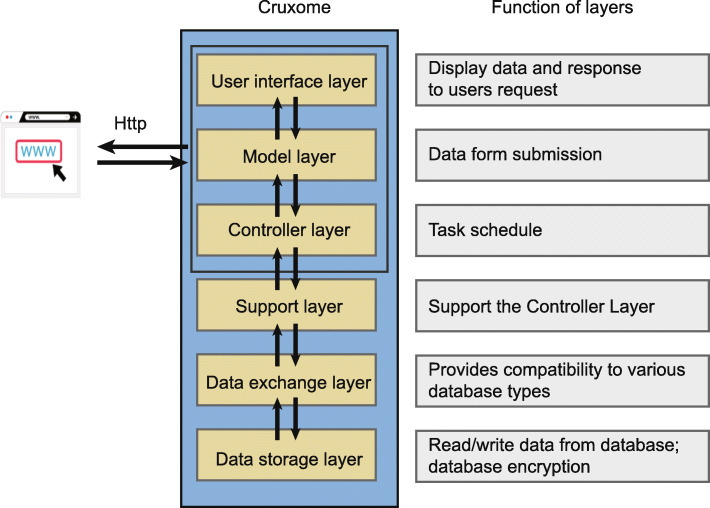
Architecture of Cruxome. The six interactive layers of Cruxome and their function are shown. Users can access Cruxome via a modern browser. The User Interface Layer, Model Layer and Controller Layer are responsible for interactive presentation to users; Data Exchange Layer provides compatibility to various database types and software, and the Data Storage Layer is responsible for data read/write operation and data security

Cruxome was designed with a user-friendly interface and developed based on a Browser/Server style to facilitate easy access and to minimize incompatibility with different computer operating systems. Cruxome runs in Docker mode (https://www.docker.com/) which is a standard unit of software that packages up code and all its dependencies so the application runs quickly and reliably from one computing environment to another. Thus, Cruxome can easily be deployed on either a cloud server (for example Amazon Web Services, Microsoft Azure) or on a local server. To enhance the functionality of Cruxome, improve efficiency and simplify code maintenance, a layered pattern was used in the basic architecture of Cruxome (Fig. 1). Cruxome consists of six sublayers: a user interface layer (UIL), a model layer (ML), a controller layer (CL), a support layer (SL), a data exchange layer (DEL) and a data storage layer (DSL). UIL, ML and CL provide the interactive and data presentations to users; SL provides support to CL; DEL provides compatibility to various database types and a connection to laboratory information management systems (LIMS) and other software and DSL is responsible for read/write data from database (MySQL as default, https://www.mysql.com/) and for storage of the information.

Minimum requirements of Cruxome (available on all modern computers):

- A modern browser (Chrome, FireFox, Safari or Edge).
- A 24-core server with 64G memory, 1T hard disk.
- An internet or intranet connection of 10Mbit.

## Results

### Cruxome pipeline

**Fig. 2:**
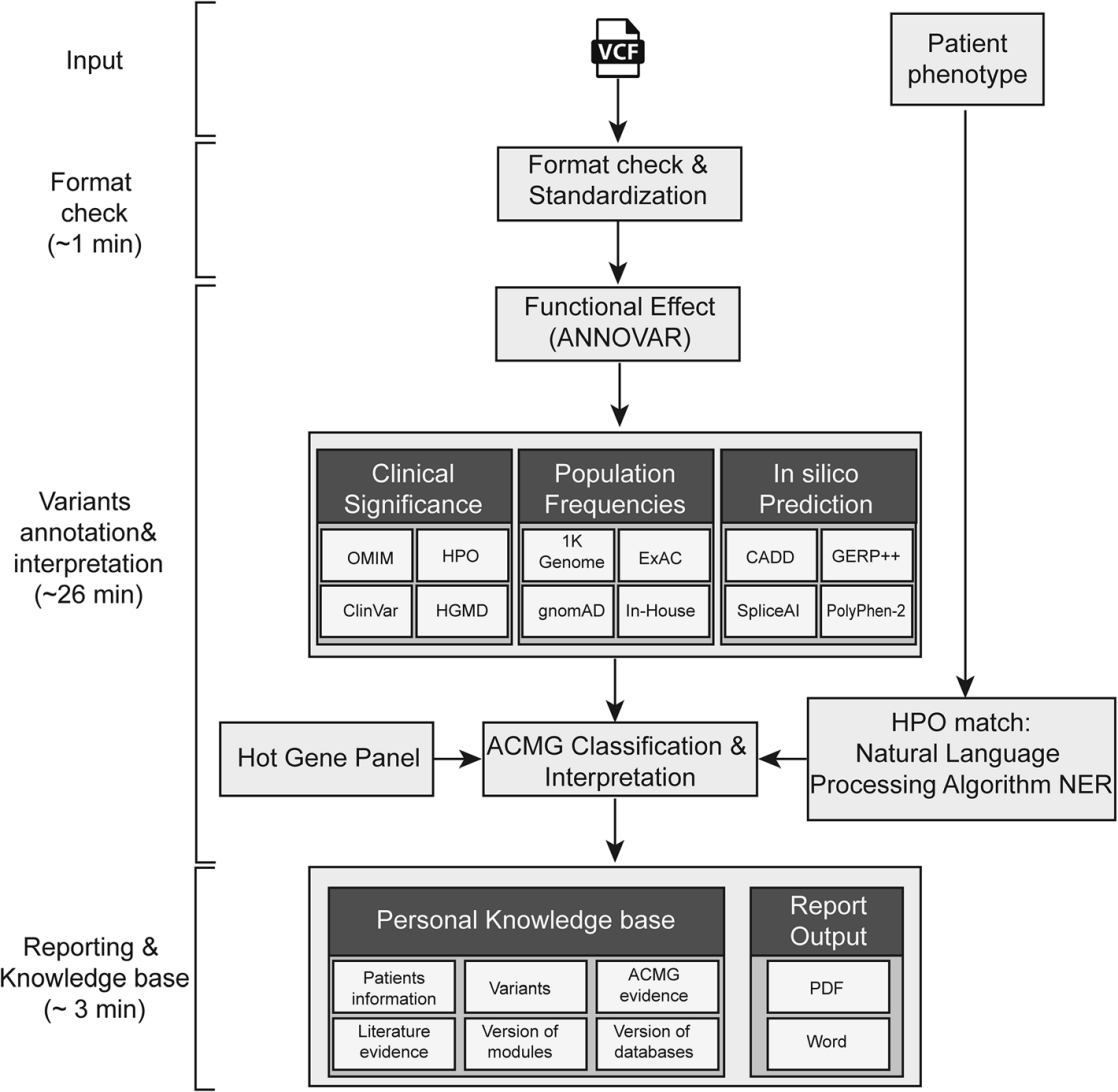
Schematic representation of Cruxome workflow. Typical Cruxome workflow contains four steps (input, format check, variants annotation and interpretation, and reporting and knowledge base), and time consumption to perform each step is indicated. After uploading a VCF file, Cruxome executes the VCF file format check in “Format check” step. Sequential “Variants annotation and interpretation” step is then launched to annotate and interpret the variants using various tools and databases. By using Natural Language Processing Algorithm NER, the hot gene panel and multiple databases, Cruxome performs integrated analyses that interpret and score variants according to ACMG guidelines. The report of the analysis is then exported in a PDF or Word format, and the personal knowledge base is automatically updated to store all the interpretation information. From sample input to report, the whole process takes approximately 30 min. OMIM: Online Mendelian Inheritance in Man, a comprehensive, authoritative compendium of human genes and genetic phenotypes; HPO: Human Phenotype Ontology, provides standardized vocabulary of phenotypic abnormalities encountered in human disease; ClinVar: a publicly available database that aggregates information about sequence variation and its relationship to human health; HGMD: Human Gene Mutation Database, represents all known (published) gene lesions responsible for human inherited disease; 1 K Genome: data from 1000 Genomes Project; ExAC: Exome Aggregation Consortium; gnomeAD: Genome Aggregation Database; In-House: genomic database of Berry Genomics Co., Ltd.; CADD: Combined Annotation Dependent Depletion, integrates multiple annotations into one metric; GERP++: Genomic Evolutionary Rate Profiling; SpliceAI: A deep learning-based tool to identify splice variants; PolyPhen-2: predicts possible impact of an amino acid substitution on the structure and function of a human protein

The overall workflow of Cruxome is shown in Fig. 2. The workflow of Cruxome commences with uploading of VCF files listing the genetic variants identified from gene panel or WES data and, uploading of the phenotypic records of each patient. Next, Cruxome performs variant annotation, phenotype processing and interpretation, and then generates a standardized report summarizing the candidate genetic variants, and provides conclusions and relevant references (PDF or Word format). A user manual file for step by step instruction of how to use the Cruxome software is available for download on the Cruxome website.

### Typical application scenario

After login to Cruxome, the user first creates the patient’s record with detailed information about phenotype, age, family relationship or directly imports the records from existing patient databases. Secondly, the VCF files are uploaded. Cruxome supports all of the VCF file formats that meet VCF 4.2 standard or higher, and supports both the GRCh37 (hg19) and GRCh38 reference genomes. Cruxome then automatically checks file formats and standardizes the files.

After checking the patient information and VCF file format, Cruxome then launches its annotation module. For the most comprehensive evaluation and interpretation of variants, Cruxome integrates multiple databases, including sequence databases for gene functional information, population databases for calculation of variants allele frequencies and disease databases to define clinical significance and phenotype relationships relevant to disease phenotype. Multiple tools are then applied to evaluate the effect of variants on protein function and to finally generate a variant score [37].

Next, Cruxome uses a natural language processing algorithm NER to transform clinical diagnosis records to HPO standard format (Fig. 2). By using our newly developed algorithm, Cruxome automatically performs the variant interpretation and clinical classification by combining the phenotypic diagnosis description and the hot gene panel according to American College of Medical Genetics and Genomics and the Association for Molecular Pathology (ACMG/AMP) guidelines [38]. At the end of the process, Cruxome generates outputs of variant interpretation results with corresponding evidence ordered by pathogenic criterion. Users can further review the interpreted variants, combine more clinical information if required and then generate a clinical report summarizing the relevant genetic variants, conclusions and references (PDF or Word format) (Fig. 2).

### Management of your own knowledge base

Once variant interpretation is complete, Cruxome automatically updates your personal knowledge base and stores all the information generated during the interpretation process, including candidate variants, ACMG evidences, literature and versions of modules and databases, thus making the interpretation decisions traceable (Fig. 2). Personal knowledge base dramatically facilitates data tracking, data management and re-interpretation variants using updated databases. Users can also manually modify or update literature records in their own knowledge base, including the clinical level of variants or other fields. When the same variants are again found following the analysis of new samples, the variants are automatically highlighted showing the information from previous records and thus provides users greater confidence with the case at hand.

### User case demonstration

**Fig. 3:**
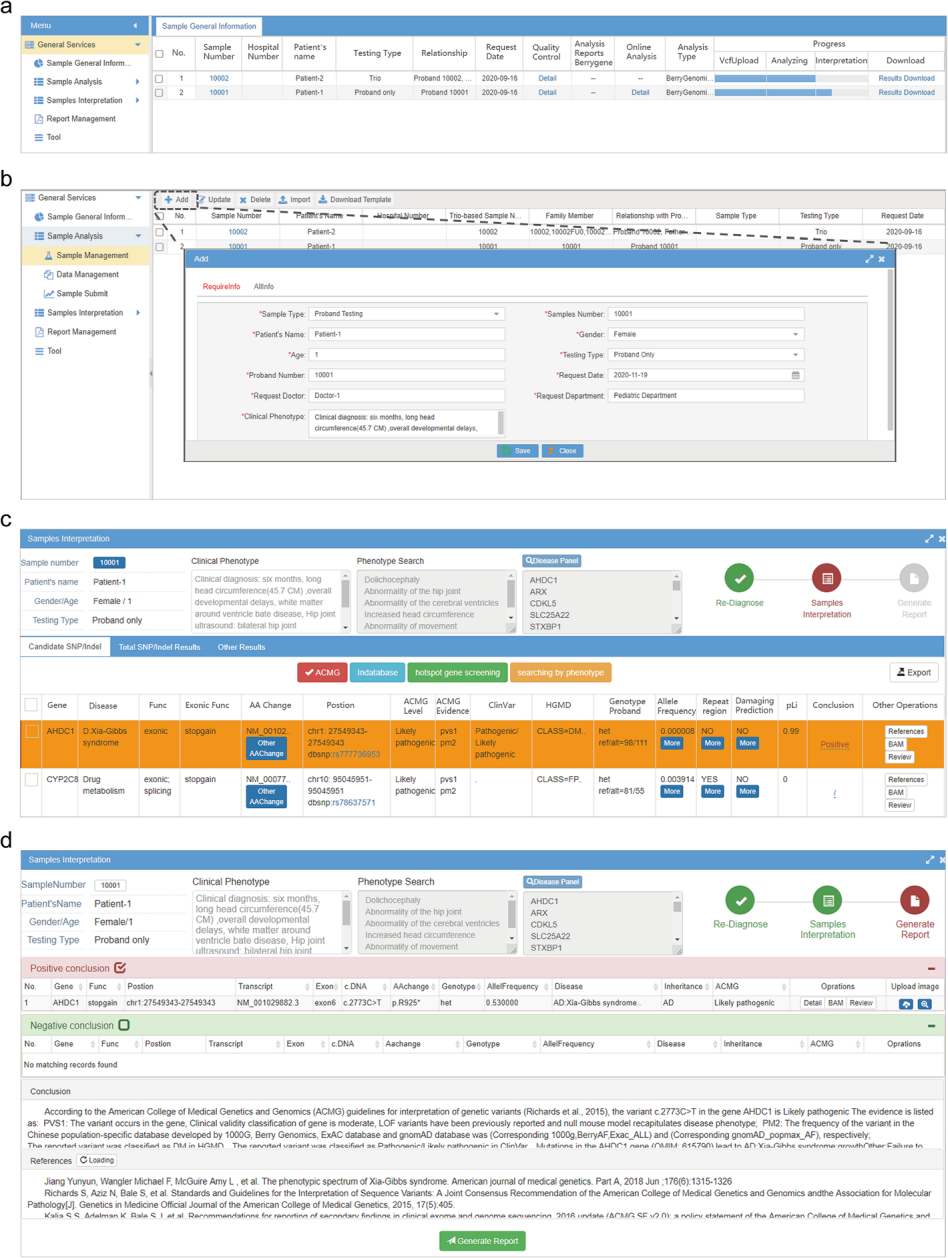
Interface of Cruxome. **A**. Home page of Cruxome, which shows a module list (left panel) and overview of all the information of samples under user’s account (right panel). Users enter the submodule by clicking the corresponding text in the left panel. **B**. To perform an interpretation, click “Add” button in “Sample Management” module, and input the patient’s information in the pop-out window. **C**. After Cruxome finishes interpretation process, a detailed list of variants with annotation and ACMG classification is produced. Users can review the interpretation of variants and examine the literature and bam file by clicking corresponding button. Candidate variants could be marked as “Positive” or “Negative” by clicking “/” in conclusion column. **D**. After entering the “Generate Report” page, users can easily export a clinically standardized report with the inclusion of all supported knowledges by choosing the “Positive conclusion” or “Negative conclusion” checkbox. Report can be found in “Report Management” module

A representative proband-only case is presented to demonstrate the functionality of the Cruxome pipeline (Fig. 3). The clinical diagnosis of the six-month-old proband was “decreased fetal movement in the prenatal period and increased head circumference (45.7 cm), global developmental delay, periventricular leukomalacia, hip dysplasia, motor deterioration and impaired pursuit initiation and maintenance post birth”. After login to Cruxome, the home page is loaded (Fig. 3A). The left panel of home page shows the modules of Cruxome whereas the right panel shows the list of patient records. After clicking the “Add” button in Sample Management module, patient’s information such as name, gender, age, clinical phenotype needs to be entered into the pop-up window (Fig. 3B). The VCF file is then uploaded (click “import” button), and Cruxome automatically performs variant interpretation. The progress of VCF uploading, analyzing and interpretation can be visualized in real time by the progress bar on the home page (Fig. 3A). The final interpretation results can be accessed in the “Sample Interpretation” module (Fig. 3C). Supporting information about variants or interpretation can be examined or reviewed by clicking the corresponding button. If candidate pathogenic gene variants are found (*AHDC1* gene in this case), users should mark the corresponding variants as “Positive” in the conclusion column (Fig. 3C). By clicking the “Generate Report” icon in upper-right of interpretation results (Fig. 3B), the generate report page will be loaded (Fig. 3D). By choosing the “Positive conclusion” or “Negative conclusion” selection box, variants, references and an automated conclusion will be displayed in corresponding section (Fig. 3D). In this example case, Cruxome successfully identified a pathogenic variant (NM_001029882.3: c.2773 C > T: p.R925*) in the *AHDC1* gene, which has been reported to be responsible for autosomal dominant Xia-Gibbs syndrome [39]. Users can simply export a standardized clinical report by clicking the “Generate Report” button below (Fig. 3D). The new report can be accessed in Report Management module.

In another representative trio-family case, the clinical diagnosis of the proband was hyperhomocystinemia, methylmalonic acidemia, anemia, megaloblastic anemia, proteinuria, occult blood, feeding difficulties. Cruxome successfully identified a likely pathogenic (NM_015506.2: c.80 A > G: p.Q27R) and a pathogenic (NM_015506.2: c.217 C > T: p.R73X) variant in the *MMACHC* gene, which is responsible for methylmalonic aciduria and homocystinuria [40] (Supplemental Table 1). The proband was a compound heterozygote for variants p.Q27R and p.R73X whereas the father and mother were confirmed to be heterozygous for the respective variants.

### Extra Tools

**Fig. 4:**
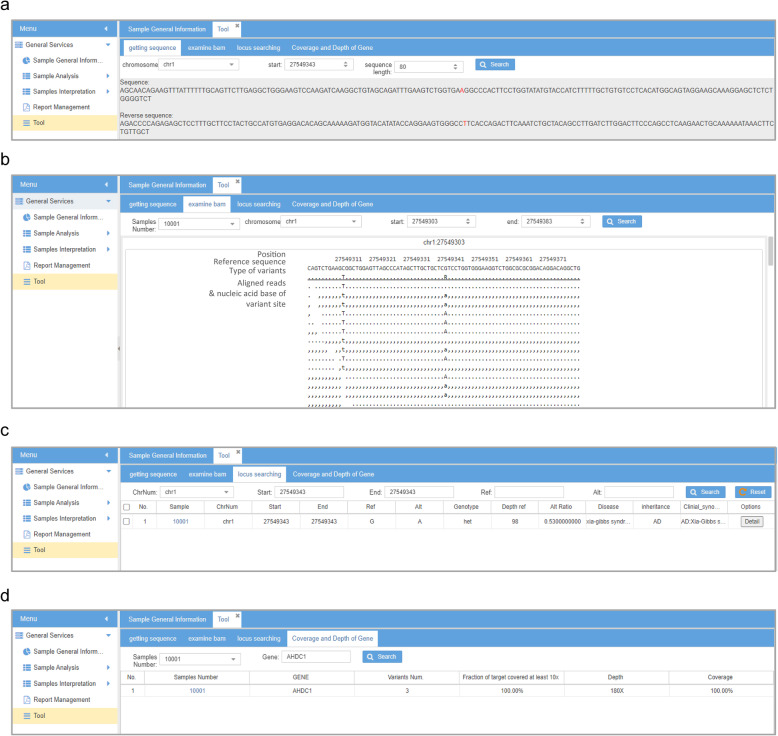
Extra tools Interface of Cruxome. **A**. “Getting sequence” tools: displays flanking sequence of a given site in the reference genome. Nucleotides marked with red indicates the query position. **B**. “Examine bam file” tool: displays reads aligned to the reference genome. The position, reference sequence and the type of variants are shown in the above three rows. Altered nucleotides in aligned reads are shown; nucleotides in reads with no change compared with the reference sequence are indicated as “,” or “.”. **C**. “Locus search” tool: calculates the frequency of variants in a given region in all samples in the personal knowledge base. **D**. The “Gene coverage and depth” tool: examines the number of variants, fraction of targets covered, coverage, depth and coverage of a given gene in all samples under the users account

Cruxome also provides other useful tools to help clinical geneticists visualize their data. First, the “getting sequence” tool can display DNA sequence of a given region (Fig. 4A). Second, the “examine bam file” tool can be used to schematically display NGS reads that aligned on the reference genome (Fig. 4B). Third, the “locus searching” tool can be used to calculate frequency of variants in all samples in the personal knowledge base (Fig. 4C). Lastly, the “gene coverage and depth” tool can search coverage, depth and the number of variants of a given gene in all samples (Fig. 4D).

### Update and version options

Cruxome is frequently updated to incorporate the latest clinical genetic research findings with options for adding new algorithms and new annotation sources and analysis modules. Benefitting from version updates, Cruxome users can easily re-analyze cases stored in the knowledge base, and potentially identify novel pathogenic variants.

### Comparison of Cruxome with other software

A range of commercial software has been reported to perform variant annotation and interpretation [26, 41–43]. Compared with above mentioned software, Cruxome offers unique advantages (Table 1). Firstly, it facilitates (i) transformation of colloquial description of phenotype to standard HPO vocabulary using a natural language processing algorithm (NER), (ii) automatic variant annotation and interpretation which greatly reduces the workload of users and (iii) export of a standard clinical report summarizing the relevant genetic variants, conclusions and references. However, in the current version of Cruxome, only variants from WES and panel data are supported, and file format of variants is restricted to standard VCF format. This limitation prevents its usages in annotating and interpretating variants from whole genome sequencing (WGS), and reduces flexibility of input files. Accordingly, further development of Cruxome is planned to include modules for annotation and interpretation of WGS variants, and modules that accept various files that contain structured records of variants (e.g. Excel or txt format) as input.

**Table 1:** Functional comparison of different software

Software	Cruxome	Seqmax	QIAGEN	TGex	Seave	InterVar
**Input file**	VCF	Fastq	VCF	VCF	VCF	VCF
**Variants**	SNV, Indel	SNV, Indel	SNV, Indel	SNV	SNV, Indel, CNV, SV	SNV, Indel
**Run mode**	Automatic	Manual	Automatic	Manual	-	Automatic
**Supports Chinese phenotype search**	YES	YES	NO	YES	NO	NO
**Phenotypic semantic analysis**	YES	NO	NO	NO	NO	NO
**Report**	Variants and clinical interpretation	Variants	Variants	Variants	NO	NO
**Database build**	YES	NO	NO	NO	NO	NO

## Conclusions

By using in-house algorithms and multiple databases, Cruxome can effectively perform variant annotation and interpretation. A user-friendly interface combined with a natural language processing algorithm NER makes Cruxome easy-to-use and importantly, users do not need to change their phenotype descriptions that they write in clinical diagnosis records. Although Cruxome is designed for users with less bioinformatics knowledge, others with a more solid grounding in bioinformatics can also use Cruxome in a more convenient and time-saving way. These features make Cruxome more versatile for use by clinical geneticists and can also provide important information to genetic counselors to discuss the results with patients. Above all, Cruxome is a powerful solution for annotating and interpreting variants and for managing personal knowledge bases and, overcomes the current bottleneck of clinical geneticists spending valuable time mining and evaluating causative variants.

## Availability and requirements

Project name: Cruxome.

Project home page: http://114.251.61.49:10024/cruxome/.

Operating system(s): Platform independent.

Programming language: Java.

Other requirements: Java (version > = 1.8.1), Tomcat (version > = 8.0), Docker (version > = 18.03.1-ce), MySQL (version > = 5.7).

License: Free for academic and research use.

## Supplementary information

**Additional file 1.**

## Data Availability

The example used in this paper (Fig. 3 B and D, Supplemental Table 1) is available in the free trial account as a demonstration case.

## Abbreviations

NGS: next-generation sequencing
SNVs: single nucleotide variants
InDels: insertions or deletions
CNVs: copy number variants
HPO: human phenotype ontology
ACMG/AMP: American College of Medical Genetics and Genomics/ Association for Molecular Pathology
WES: whole exome sequencing
EHR: electronic health records
NER: natural language processing algorithm
VCF: variant call format
MAF: minor allele frequency
