# Supplementary material for: Cruxome: a powerful tool for annotating, interpreting and reporting genetic variants
Source: BMC Genomics. 2021 Jun 3;22:407. doi: 10.1186/s12864-021-07728-6 (PMC8173893; doi:10.1186/s12864-021-07728-6)
Supplement: Supplementary file 1 — Additional file 1. [file 12864_2021_7728_MOESM1_ESM.doc]

**Supplemental Table 1:** A trio-family case used in Cruxome annotation and interpretation.

| **Gene** | **Position** | **Exon** | **HGVS** | **Effect** | **Variant status** | **ACMG** | **Disease description** |
| --- | --- | --- | --- | --- | --- | --- | --- |
| *MMACHC* | chr1:45500412-45500412 | exon1 | NM_015506.2: c.80A>G: p.Q27R | Nonsynonymous SNV | Patient：Het  Father：Het  Mather：Wt | Likely pathogenic | Methylmalonic aciduria and homocystinuria, cblC type, AR |
| *MMACHC* | chr1:45507491-45507491 | exon2 | NM_015506.2: c.217C>T: p.R73X | Stopgain | Patient：Het  Father：Wt  Mather：Het | Pathogenic | Methylmalonic aciduria and homocystinuria, cblC type, AR |

A trio-family case. The proband was diagnosed with hyperhomocystinemia, methylmalonic acidemia, anemia, megaloblastic anemia, proteinuria, occult blood and feeding difficulties. Cruxome successfully identified a likely pathogenic variant c.80A>G: p.Q27R and a pathogenic variant c.217C>T: p.R73X in *MMACHC* gene. Abbreviations: *MMACHC*, metabolism of cobalamin associated C; HGVS, variant nomenclature by human genome variation society; Effect, effect of variant; Het: heterozygous nucleotide; Wt: wildtype nucleotide; ACMG: American College of Medical Genetics and Genomics/ Association for Molecular Pathology; AR, autosome recessive.
